# Mechanisms of semantic composition in older adults: control, semantic processing, and imagery

**DOI:** 10.3389/fpsyg.2026.1809368

**Published:** 2026-05-14

**Authors:** Heather Bruett, Xiaoxi Qi, Marc N. Coutanche

**Affiliations:** 1 Department of Psychology, University of Pittsburgh, Pittsburgh, PA, United States; 2 Learning Research and Development Center, University of Pittsburgh, Pittsburgh, PA, United States

**Keywords:** aging, conceptual combination, individual differences, semantic composition, semantic memory

## Abstract

**Introduction:**

Semantic composition is the ability to create meaningful novel concepts from familiar concepts. This can be achieved through a set of cognitive abilities that vary across individuals. As aging affects semantic networks in complex ways, examining how older adults experience the process of combining concepts can clarify which cognitive abilities contribute to maintaining flexible conceptual thinking.

**Methods:**

In this study, 87 healthy older adults and a comparison group of 83 younger adults completed a semantic composition task, along with tasks measuring individual variation in cognitive processes hypothesized to underlie semantic composition: semantic processing, cognitive control, divergent and convergent creative thinking, and visual imagery.

**Results:**

In older adults, ease of semantic composition (in the form of conceptual combination) was predicted by cognitive control abilities, particularly when combining ambiguous word pairs that require resolving competing interpretations. In addition, older adults with stronger semantic processing and more vivid visual imagery found conceptual combination easier, with visual imagery especially benefiting attributive combinations. In younger adults, by contrast, ease of combining was more selectively related to cognitive control, with less evidence that semantic processing or imagery contributed in the same way.

**Discussion:**

These findings demonstrate that the experienced ease of semantic composition in older adults reflects contributions from multiple cognitive mechanisms, varying by combination type, to maintain the capacity for conceptual flexibility.

## Mechanisms of semantic composition in older adults: control, semantic processing, and imagery

Semantic composition, also known as “conceptual combination”, refers to the process by which humans create novel meanings by combining existing concepts. It is a skill important for flexible communication and creative thought, underlying a wide range of cognitive activities, such as language understanding, problem solving, and learning. Compositions can take different forms: *attributive* compositions derive meaning from a particular feature of the concepts (e.g., *bullet train* might be a fast train; Coutanche et al., 2020; Estes, 2003), while *relational* compositions depend on a relationship between the concepts (e.g., *yarn truck* might be a truck containing yarn). Some compositions are ambiguous, placing varying demands on semantic memory and cognitive control. Related work in the associative memory literature has used compound-like word pairs to examine whether some pairings can be encoded more holistically, often described as unitized representations (e.g., Haskins et al., 2008; Quamme et al., 2007).

Because novel combinations often have multiple valid interpretations, objectively assessing the combination process is difficult. However, the metacognitive experience of felt ease reflects real-time monitoring of processing fluency (Hertzog and Dunlosky, 2011; Koriat, 2007), providing a window into how individuals navigate semantic composition.

Aging presents a paradox for semantic composition. Older adults possess broader vocabularies and richer conceptual networks (Dubossarsky et al., 2017), yet aging is associated with declines in cognitive control and processing efficiency that may impair flexible concept combination (Lucas et al., 2019; Shafto and Tyler, 2014). Reduced inhibitory control can lead to “hyper-binding”—excessive associations and heightened interference vulnerability (Campbell et al., 2010). Importantly, metacognitive monitoring often remains preserved with age (Kavé and Halamish, 2015; Murphy and Castel, 2024), making subjective ease a sensitive indicator of how cognitive mechanisms coordinate during composition.

We investigated four cognitive mechanisms that may relate to semantic composition in older adults. *Semantic processing*, the ability to navigate conceptual space, draws heavily upon the brain's semantic network (Coutanche et al., 2020; Law et al., 2026). *Cognitive control* guides retrieval by modulating connections between semantic hubs and perceptual regions (Rogers et al., 2015; Gao et al., 2023); reduced inhibitory control can lead task-irrelevant information to interfere with processing (Hasher and Zacks, 1988). We also examined *creativity*, including divergent thinking (flexibly recombining information; Guilford, 1967) and convergent thinking (narrowing to a best solution; Cropley, 2006). Finally, *visual imagery* may support composition through perceptual simulation (Wu and Barsalou, 2009), particularly for attributive combinations requiring feature integration.

The present study investigates how these individual differences predict subjective ease of semantic composition in older adults and how relationships vary by combination type. Individual differences in these mechanisms may help resolve the aging paradox, as some individuals maintain performance through compensatory processes (Reuter-Lorenz and Park, 2014; Stern, 2012).

## Methods

### Participants

We recruited 100 older adults through Prolific and 100 younger adults through the university's student subject pool. Participants were required to have the United States as their native country, English as a first language, and English as their most proficient language. One older and 15 younger adults did not meet language criteria and were excluded from the study. After exclusions for language criteria and task engagement (see Results), the analyzed sample consisted of 87 older adults (37 male, 50 female; M = 69.63 years, SD = 3.50) and 83 younger adults (39 male, 44 female; M = 18.55 years, SD = 0.82). Older adults were the primary sample of interest; younger adults were included as a comparison group to contextualize age-related patterns across individual difference measures and reported ease of semantic composition.

### Procedure

Participants completed a conceptual combination task with 60 word-pairs (20 attributive, 20 relational, and 20 ambiguous); classification was determined in an independent norming study based on participants' produced definitions and independent coding (see Text S1 for details). On each trial, participants viewed the word pair for 2 sec, then rated how well they could define the combination on a scale of 1 (not well at all) to 7 (very well) for 4 sec. Participants were not asked to explicitly provide definitions for each combination, so these ratings reflect metacognitive ease judgments, rather than definition accuracy. We also validated ease judgments against difficulty ratings from the independent norming sample (Text S1). Following the combination task, participants completed a cognitive battery in a randomized order to measure individual differences that might predict performance on the conceptual combination task.

### Materials

Participants completed two creativity tasks. Divergent thinking was measured using the Alternate Uses Task (Guilford, 1967). Participants were asked to generate creative uses for two common objects (“brick” and “paperclip”) within 3 min total (Stevenson et al., 2020). Responses were scored using SemDis (Beaty and Johnson, 2021), which calculates semantic distance between responses and the original object. For each participant, AUT performance was calculated by taking the average semantic distance across all scored responses, such that higher values indicate larger semantic distance on average (i.e., greater creativity). Semantic distance has shown validity as an automated measure of AUT originality (Beaty and Johnson, 2021). We chose to use the average score to capture each participant's typical level of originality across the range of their responses (Beaty and Johnson, 2021; Beaty et al., 2022). Average SemDis scores were recently linked to activity in the default mode network (Bartoli et al., 2024), which is strongly associated with creativity (Luchini et al., 2025). Notably, average SemDis scores were affected by causal manipulation of default mode activity (through cortical stimulation), without affecting response fluency, consistent with average SemDis acting as a specific measure of originality in divergent thinking. Convergent thinking was assessed with the Remote Associates Task (RAT; Taft and Rossiter, 1966). Participants were presented with three cue words and required to identify a single word that linked all three cues (e.g., cottage/swiss/cake → cheese). The task consisted of 30 trials, each lasting 15 sec. Stimuli were drawn from Bowden and Jung-Beeman (2003) and verified as still familiar in the current decade through a small verification sample (*n* = 8; full age range). Performance was scored as accuracy (proportion correct).

Visual imagery vividness was assessed using the Vividness of Visual Imagery Questionnaire (VVIQ; Marks, 1973). Participants read 16 items describing visual scenes and indicated how vividly they could visualize each on a scale from 1 (Perfectly clear and as vivid as normal vision) to 5 (No image at all, you only “know” that you are thinking of the object). The questionnaire has strong psychometric properties, with a test-retest reliability coefficient of 0.74 (*n* = 68) and split-half reliability coefficient of 0.85 (*n* = 150; Marks, 1973).

Cognitive control was measured using an arrow version of the Flanker task (Ridderinkhof et al., 2021; Stoffels and Van der Molen, 1988). Participants indicated the direction of a central arrow that was flanked by either congruent (< < < < < or >>>>>) or incongruent distractors (< < > < < or >> < >>) distractors. The task consisted of 240 trials total (120 congruent, 120 incongruent). Each trial lasted 1,500ms with an inter-trial interval of 500ms. Flanker reaction times (RTs) were transformed using the Box-Cox family transformation at the group level. Incorrect trials and those with RTs deviating more than 2.5 SDs from cell means (calculated separately for each participant) were removed prior to averaging. Flanker RT and error rate difference scores were created by subtracting congruent from incongruent values, with higher values indicating greater interference (i.e., poorer cognitive control).

Semantic processing refers to the ability to successfully access semantic relationships stored in semantic memory. Here, this was assessed using a 3-alternative forced choice (3AFC) task in which participants determined which of three words was semantically related to a cue word. The task consisted of 56 trials that varied in difficulty to capture individual differences in semantic processing ability, so that some trials were easier due to greater cue-target relatedness (e.g., tennis ball-racket) and others were more challenging with lower cue-target relatedness (e.g., butterfly-eggs). Each trial lasted until the participant submitted their response, with a maximum duration of 3 sec. The task and stimuli were modified slightly for an American audience from a recent study (Sormaz et al., 2017). Performance was scored as accuracy (proportion correct). Although both RAT and this semantic processing task involve semantic relationships, the RAT places greater demands on generating convergent solutions, while the semantic processing task relies more on a recognition-based judgment, which requires recognizing and evaluating semantic relationships from presented alternatives.

### Statistical analyses

We used linear mixed-effects regression to analyze how individual differences predict ease of combining in older adults, using the lme4 package in R (Bates et al., 2015), with *p*-values for fixed effects obtained using lmerTest (Zeileis and Hothorn, 2002). Six z-scored continuous predictors were included as fixed effects: visual imagery, semantic processing, divergent thinking, convergent thinking, Flanker RT, and Flanker error rate. Combination type (attributive, relational, ambiguous) was modeled using two orthogonal contrasts: one comparing attributive vs. relational combination, and the other comparing ambiguous vs. unambiguous combinations (with relational and attributive combinations grouped together). Interactions between each individual-difference predictor and combination-type contrasts were included. Participants were modeled with random intercepts. For comparison, we also fit a parallel younger-adults model and a pooled all-age model (all-age model reported in Supplementary Text 3). Because younger-adult data included some missing values (see Results), the younger-adult and all-age models were estimated using multiple imputation with the mice package in R (van Buuren and Groothuis-Oudshoorn, 2011).

## Results

### Validation check

A one-way ANOVA on item-averaged ease ratings revealed a significant effect of combination type (*F*_(2, 57)_ = 16.33, *p* < 0.001). Ambiguous items were rated as less easy than both attributive (*t* (57) = −4.57, *p* < 0.001) and relational items (*t* (57) = −5.25, *p* < 0.001), while attributive and relational items did not differ. Ease ratings strongly correlated with difficulty ratings collected from the norming sample at the participant level (mean *r* = −0.93, *SD* = 0.16).

### Task performance

Twelve participants were excluded for poor task engagement during semantic composition (< 3 SDs below the average SD, > 33% of trials left blank), VVIQ (< 3 SDs below the average SD), semantic (< 3 SDs below the average SD, > 33% of trials left blank), and/or RAT tasks (all trials left blank). One participant was also removed from the RAT task for misunderstanding the instructions (i.e., re-typing the cues). Basic performance for each task is reported in Table 1. Younger adults showed faster Flanker performance with a higher error rate. Older adults showed higher RAT accuracy and higher ease-of-combining ratings. Semantic processing accuracy and visual imagery vividness did not differ between groups.

**Table 1:** Summary of performance on individual difference measures for both older and younger adults.

Measure	Condition	Older		Younger			
		M	SE	M	SE	*t*	*p*
Flanker RT (inconsistent—consistent)		0.13	0.01	0.07	0.01	4.57	< 0.001
	Inconsistent	6.58	0.01	6.35	0.01	—	—
	Consistent	6.44	0.01	6.28	0.01	—	—
Flanker error rate (inconsistent—consistent)		0.08	0.01	0.33	0.03	−7.36	< 0.001
	Inconsistent	0.10	0.01	0.37	0.03	—	—
	Consistent	0.02	0.01	0.05	0.03	—	—
RAT		0.59	0.01	0.51	0.01	6.04	< .001
AUT		0.95	0.00	0.96	0.00	−3.33	.001
Semantic processing		0.81	0.00	0.82	0.01	−0.77	0.440
VVIQ		3.72	0.03	3.75	0.03	−0.71	0.480
Ease of combining		5.20	0.07	4.65	0.07	5.62	< 0.001

Flanker RT means are reported after Box-Cox family power transformation. RAT reflects accuracy (0–1). AUT reflects creativity (0 = least creative, 2 = most creative). Semantic reflects average accuracy (0–1). VVIQ reflects average response (1 = most clear, 5 = least clear). Ease of combining reflects average response (1 = not well at all, 7 = very well). The t and p-values reflect the results of between-group comparisons.

### Mixed-effects model

**Figure 1:**
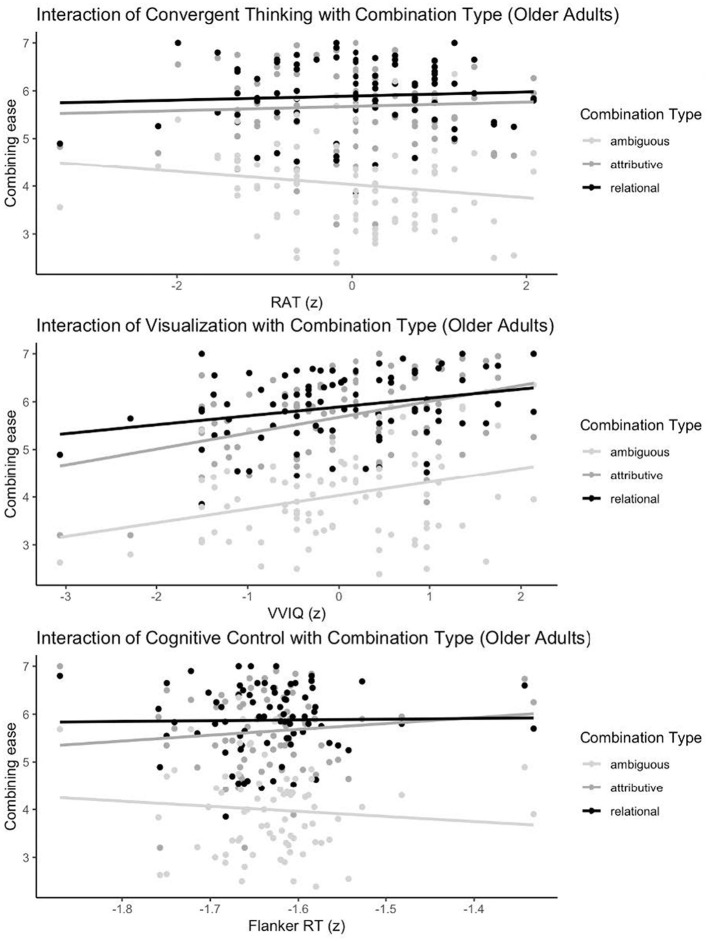
Interaction effects of individual differences and ease of combining in older adults. Flanker RTs reflect incongruent trials minus congruent trials (after z-scoring).

In older adults, a null random-intercept model reported an intraclass correlation coefficient (ICC) of 0.093. The full model (R^2^_m_ = 0.583; R^2^c = 0.856) showed that greater ease of combining was predicted by higher visual imagery (β = 0.26, *SE* = 0.08, *t* (69) = 3.15, *p* = 0.002), with an interaction with combination type (β = 0.157, *SE* = 0.08, *t* (138) = 1.99, *p* = 0.048) showing stronger effects for attributive than relational combinations. Semantic processing ability predicted greater ease (β = 0.25, *SE* = 0.09, *t* (69) = 2.94, *p* = 0.005). An interaction between convergent thinking and combination type (β = 0.22, *SE* = 0.07, *t* (138) = 3.03, *p* = 0.003) revealed a more negative relationship for ambiguous combinations compared to unambiguous ones (See Figure 1). The Flanker RT measure interacted with combination type (β = 1.88, *SE* = 0.91, *t* (138) = 2.07, *p* = 0.041), with cognitive control more strongly related to ease for ambiguous than unambiguous combinations. Neither main effect of the combination-type contrasts was significant; see full model statistics for all terms in the Supplementary Table 3.

**Figure 2:**
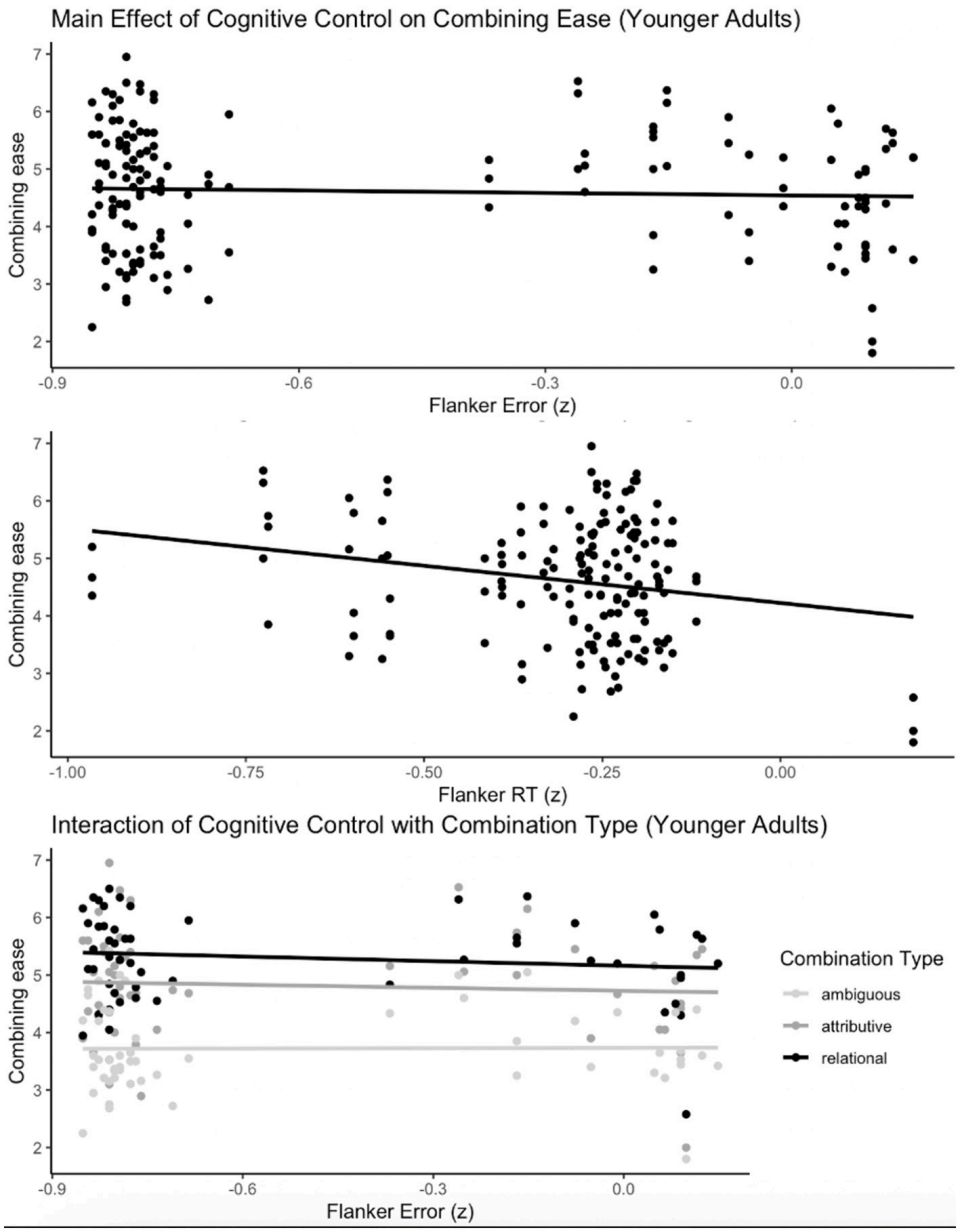
Individual difference measures predicting ease of combining in younger adults. Flanker RTs and errors reflect incongruent minus congruent trials (after all trials were z-scored).

In younger adults, a random-intercept null model reported ICC = 0.29, indicating that 29% of the variance in composition ease was between participants. The multiple imputation model showed that smaller Flanker costs were associated with greater ease: RT cost (β = −2.52, *SE* = 0.78, *t* (116) = −3.24, *p* = 0.002) and error cost (β = −0.91, *SE* = 0.33, *t* (116) = −2.76, *p* = 0.007). Flanker error × ambiguity (Unambiguous vs. Ambiguous) was also significant (β = −0.56, *SE* = 0.28, *t* (116) = −2.03, *p* = 0.044; see Figure 2). No other main effects or interactions reached significance; full model output is reported in the Supplementary Table 4.

To directly estimate the effect of age, we also fit a pooled model including age group as a fixed factor and all required interaction terms. The pooled model required multiple three-way Age × Combination type × Individual difference measures terms, substantially increasing the number of parameters. The null random-intercept model reported ICC = 0.224. The multiple imputation model did not show main effect of Age Group (β = −29.32, *SE* = 43.34, *t* (329) = −0.68, *p* = 0.499). Age Group interacted with visual imagery (β = −0.17, *SE* = 0.07, *t* (329) = −2.41, *p* = 0.016), suggesting a weaker relationship between visual imagery and composition ease in younger adults compared to older adults. Convergent thinking interacted with combination type (β = 0.17, *SE* = 0.06, *t* (329) = 2.70, *p* = 0.007), reflecting a more positive relationship between convergent thinking and composition ease for unambiguous than ambiguous pairs, averaged across age. A three-way interaction, Age Group × VVIQ × combination type, was also significant (β = −0.162, *SE* = 0.06, *t* (329) = −2.51, *p* = 0.012), indicating that the differences in composition ease between attributive and relational pairs because of visual imagery are smaller in younger adults. No other main effects, two-way interactions, or three-way interactions reached significance (see full model output in Supplementary Table 6). Because the association between cognitive control measures (Flanker RT and errors) differed by age group (younger: *r* = −0.46; older: *r* = 0.32; Supplementary Table 5), indicating distinct control strategies, pooling the groups would average over these opposite patterns (blurring age-specific effects) and make them difficult to interpret. For this reason, we focus the interpretation and discussion of our findings on the age-specific models.

## Discussion

This study examined how cognitive control, semantic processing, creativity, and visual imagery relate to subjective ease of semantic composition in older adults, while also using a younger comparison group to contextualize age-related patterns. In older adults, ease ratings strongly corresponded with independent norming difficulty ratings, confirming our metacognitive measure captures meaningful variation. In older adults, ease of combining was predicted by stronger semantic processing and visual imagery abilities, with the latter showing particular benefits for attributive combinations. Cognitive control selectively facilitated ambiguous combinations, while convergent thinking was associated with greater difficulty for these same combinations. In younger adults, by contrast, ease of combining was more selectively related to cognitive control, with less evidence that semantic processing or imagery contributed in the same way.

Cognitive control emerged as a key predictor for ambiguous items in older adults, aligning with inhibition's role in creative cognition (Abraham, 2014). This benefit may resemble cognitive control's role in metaphor comprehension (Solomon and Thompson-Schill, 2017; Holyoak and Stamenković, 2018), where inhibition helps prioritize contextually relevant properties. While older adults often struggle to inhibit irrelevant literal text, they maintain this skill for metaphor processing (Hasher and Zacks, 1988), suggesting similar mechanisms may support semantic composition.

Semantic processing positively predicted composition ease across all combination types, confirming its foundational role in supporting semantic composition in older adults. As semantic networks become less connected with age (Cosgrove et al., 2021; Dubossarsky et al., 2017), individual differences in navigating those networks may become increasingly essential, suggesting preserved semantic processing could serve as a protective factor.

The lack of relationship between divergent thinking and composition ease was initially surprising. One likely explanation is time constraints: older adults may require additional time to engage divergent strategies (Fusi et al., 2021). Similar logic may explain why convergent thinking was associated with greater difficulty for ambiguous combinations. Under time pressure, older adults' reliance on convergent thinking, which prioritizes single solutions, may impair their performance for items benefiting from multiple interpretations.

Visual imagery positively predicted ease of composition in older adults, with the strongest effect for attributive combinations that rely on perceptual feature manipulation. One possible explanation is that, in older adults, visual imagery may become more important for semantic composition because it offers perceptual simulation to support composition as other relevant cognitive functions become less efficient with age. This could be especially helpful for attributive combinations, where meanings often depend on mentally representing a salient feature of one concept as modifying another (e.g., bullet train as a fast train). In contrast, relational combinations might rely less on simulating features and more on identifying a plausible relation between the two concepts (e.g., mountain snake as a snake that is found on mountains).

These findings reveal that multiple cognitive substrates contribute to semantic composition ease in older adults, consistent with a compensatory framework. Relative to the younger comparison group, older adults showed a broader pattern of associations, with semantic processing and visual imagery contributing in addition to cognitive control. As semantic networks become less efficient with age (Cosgrove et al., 2021), individuals may increasingly rely on preserved abilities –cognitive control, visual imagery, semantic processing– to maintain performance. The specific compensation pattern depends on both individual strengths and combination type demands.

### Limitations

Our focus on within-age associations limits conclusions about age-related differences. Internet-using older adults from Prolific tend to have higher education and cognitive functioning than the general population (Berner et al., 2015). However, the relation between Flanker RT and Flanker error relation differed by age, which is consistent with the well-studied speed-accuracy tradeoff (for review, see Heitz, 2014) and age-related shifts toward more accuracy-focused response strategies (e.g., Ratcliff et al., 2001; Starns and Ratcliff, 2010). Pooling across these opposing patterns would risk averaging away age-specific effects though for transparency, we report the all-age model in the Supplementary Materials. We acknowledge that although the semantic processing task has been associated with individual differences in hippocampal connectivity (Sormaz et al., 2017; Vatansever et al., 2021), it has less precedent in the literature as an established individual difference measure relative to the other measures used in this study. We also note that participants did not provide combination definitions, limiting objective scoring of their performance; however, given multiple valid interpretations for most combinations, subjective ease may be a more appropriate measure to capture individuals' experience of the combination process.

In summary, semantic composition in older adulthood draws on distinct cognitive mechanisms depending on combination type. Cognitive control facilitates resolution of ambiguous combinations, semantic processing provides foundational support across combination types, and visual imagery particularly supports attributive combinations. Understanding individual cognitive strengths has implications for maintaining communicative and creative abilities in later adulthood.

## Data Availability

The raw data supporting the conclusions of this article will be made available by the authors, without undue reservation.
