# Supplementary material for: Mechanisms of semantic composition in older adults: control, semantic processing, and imagery
Source: Front Psychol. 2026 May 14;17:1809368. doi: 10.3389/fpsyg.2026.1809368 (PMC13216000; doi:10.3389/fpsyg.2026.1809368)
Supplement: Supplementary file 1 [file Supplementary_file_1.DOCX]

**Supplementary Materials**

**Text S1. Norming Study**

Word pairs in the study were selected from a norming study. The norming study first identified word pairs used in past investigations of semantic composition for specific combination types. Attributional word pairs (*n* = 48) were selected from Estes, 2003; relational pairs (*n* = 58) from Estes, 2003 and Wisniewski & Love, 1998; and ambiguous pairs (*n* = 82) from Gagné & Shoben, 1997 and Kenett & Thompson-Schill, 2017. From these full lists, eight pairs were removed for containing compound words (e.g., *sleeping-pill personality*) and fourteen were removed for having matching taxonomic categories between the words. All word pairs containing words with concreteness levels below a 4 according to the Brysbaert Concreteness Ratings (*n* = 43) were removed, as were any remaining duplicate word pairs (*n* = 4). Ultimately, this left 34 attributive, 41 relational, and 48 ambiguous pairs to be used in the norming study.

In the norming study, we collected data from 60 participants through Prolific (https://prolific.co/). Each participant viewed a random selection of 30 pairs (of the 123 selected earlier). For each pair, participants were asked to provide their preferred definition for the combination, indicate their ease for providing the definition using a 1 to 7 Likert scale to indicate their agreement with two statements (i.e., “I had trouble thinking of a definition for this word combination.”; “I feel that multiple definitions could apply to this word combination equally well.”), and finally to indicate if they were unfamiliar with either word. Finally, all norming participants completed a basic demographics questionnaire. To match our norming study sample with that of the main study, participants who indicated that they had a neurological disability, intellectual or learning disability, and/or attentional disorder, as well as participants who did not meet our language requirements, were removed, giving 55 participants in the final norming study sample.

Three independent raters categorized the participants’ definitions as attributional, relational, neither, impossible to determine, or unsure. Raters were trained on identifying relational and attributional combinations in advance. They categorized definitions as “attributional” if one dominant attribute or feature from the modifier noun was applied to the head noun, and “relational” if a relationship was formed between the nouns. “Neither” was used to categorize valid definitions where words were combined without attributional or relational techniques. For example, if a definition morphed the nouns (e.g., describing an *elephant ant* as a medium-sized creature with antennae and a tail) or used the second noun to modify the first (e.g., describing an *elephant ant* as a small elephant), it was classified as “neither.” Definitions were categorized as “impossible to determine” if they did not have enough detail for another classification (e.g., participant only wrote one word, wrote nonsense, etc.). Definitions categorized as “unsure” were re-categorized to one of the other definitions by an independent final judge. After ratings, final categorizations were determined in a winner-takes-all manner, meaning that definitions that were given the same rating by at least 2 of the independent raters were categorized according to their judgment.

A total of 60 nouns were selected (20 attributional, 20 relational, 20 ambiguous). The final stimuli list can be seen in Table S1. All attributional and relational nouns had at least 75% of definitions categorized as attributional or relational, respectively (see Table S2). Ambiguous nouns were selected if they were categorized as below 60% attributional and relational.

**Table S1.**

*Final list of stimuli and their categorization.*

| **Attributional** | **Relational** | **Ambiguous** |
| --- | --- | --- |
| leech boyfriend | rugby shoes | centipede table |
| chocolate clay | yarn truck | onion bus |
| pillow lips | concrete fountain | copper horse |
| lemon paint | grill steak | cracker wall |
| finger tree | patio cigarette | iron fist |
| rock bagel | car photographer | trash house |
| bullet train | burrito stain | grease fish |
| fire coffee | rodeo magazine | sap toy |
| vampire insect | book string | olive signals |
| sedative voice | tissue alcohol | cake confetti |
| octopus chair | bowling sweater | liquor keyboard |
| thunder applause | floor television | attic leg |
| silk hair | prisoner graffiti | picture soup |
| rocket sprinter | scalpel incision | sink tub |
| molasses traffic | mountain snake | alligator mouth |
| strawberry ink | microwave sandwich | air lakes |
| piranha lawyer | boxing bruise | wreath nerves |
| canary crayon | student artwork | cardboard lotion |
| balloon pregnancy | pancake spatula | melon planet |
| butter grip | song court | pudding lamp |

*Note.* List of all nominal pairs.

**Table S2.**

*Average categorization as attributional or relational combinations.*

|  | | Ratings from independent rater | |
| --- | --- | --- | --- |
|  |  | Attributional | Relational |
| Each stimulus condition | Attributional | 90.1% | 5.0% |
|  | Relational | 2.2% | 95.3% |
|  | Ambiguous | 36.4% | 43.0% |

*Note.* Ratings from independent raters for each stimulus condition.

**Table S3. Full statistical model in older adults.**

|  | **Estimate** | ***SE*** | ***df*** | ***t*** | ***p*** |  |
| --- | --- | --- | --- | --- | --- | --- |
| (Intercept) | -7.69 | 30.50 | 69 | -0.25 | 0.802 |  |
| VVIQ | 0.26 | 0.08 | 69 | 3.15 | 0.002 | ** |
| Semantic | 0.25 | 0.09 | 69 | 2.94 | 0.005 | ** |
| AUT | -0.63 | 1.68 | 69 | -0.38 | 0.706 |  |
| RAT | -0.10 | 0.09 | 69 | -1.17 | 0.245 |  |
| Flanker RT | -0.92 | 1.11 | 69 | -0.83 | 0.409 |  |
| Flanker Error Rate | 0.58 | 0.39 | 69 | 1.48 | 0.143 |  |
| Ambiguity (Unambiguous vs. Ambiguous) | 42.30 | 25.07 | 138 | 1.69 | 0.094 |  |
| Unambiguous (Attributive vs. Relational) | 23.59 | 28.95 | 138 | 0.82 | 0.417 |  |
| VVIQ x Ambiguity | -0.02 | 0.07 | 138 | -0.25 | 0.806 |  |
| VVIQ x Unambiguous | 0.16 | 0.08 | 138 | 1.99 | 0.048 | * |
| Semantic x Ambiguity | -0.02 | 0.07 | 138 | -0.25 | 0.802 |  |
| Semantic x Unambiguous | -0.02 | 0.08 | 138 | -0.20 | 0.846 |  |
| AUT x Ambiguity | 2.06 | 1.38 | 138 | 1.50 | 0.137 |  |
| AUT x Unambiguous | 1.23 | 1.59 | 138 | 0.78 | 0.440 |  |
| RAT x Ambiguity | 0.22 | 0.07 | 138 | 3.03 | 0.003 | ** |
| RAT x Unambiguous | 0.01 | 0.08 | 138 | 0.13 | 0.895 |  |
| Flanker RT x Ambiguity | 1.88 | 0.91 | 138 | 2.07 | 0.041 | * |
| Flanker RT x Unambiguous | 0.76 | 1.05 | 138 | 0.73 | 0.469 |  |
| Flanker Error Rate x Ambiguity | -0.31 | 0.32 | 138 | -0.97 | 0.334 |  |
| Flanker Error Rate x Unambiguous | 0.32 | 0.37 | 138 | 0.85 | 0.398 |  |

*Note.* Results of regression model of individual differences predicting ease of conceptual combination in older adults. Model: lmer(ease ~ 1 + vviq_z + semantic_z + aut_z + rat_z + flanker_rt_z + flanker_err_z + comboType + vviq_z:comboType + semantic_z:comboType + aut_z:comboType + rat_z:comboType + flanker_rt_z:comboType + flanker_err_z:comboType + (1|id), data = indivDiff_data)

**Table S4. Full statistical model in younger adults.**

|  | **Estimate** | ***SE*** | **statistic** | ***df*** | ***p*** |  |
| --- | --- | --- | --- | --- | --- | --- |
| (Intercept) | -75.53 | 78.17 | -0.97 | 116 | 0.336 |  |
| VVIQ | -0.10 | 0.11 | -0.95 | 116 | 0.345 |  |
| Semantic | 0.10 | 0.15 | 0.71 | 116 | 0.478 |  |
| AUT | -3.10 | 3.07 | -1.01 | 116 | 0.315 |  |
| RAT | -0.03 | 0.13 | -0.21 | 116 | 0.832 |  |
| Flanker RT | -2.52 | 0.78 | -3.24 | 116 | 0.002 | ** |
| Flanker Error Rate | -0.91 | 0.33 | -2.76 | 116 | 0.007 | ** |
| Ambiguity (Unambiguous vs. Ambiguous) | 23.48 | 66.02 | 0.36 | 116 | 0.723 |  |
| Unambiguous (Attributive vs. Relational) | 56.10 | 76.23 | 0.74 | 116 | 0.463 |  |
| VVIQ x Ambiguity | -0.07 | 0.09 | -0.75 | 116 | 0.456 |  |
| VVIQ x Unambiguous | -0.17 | 0.10 | -1.59 | 116 | 0.114 |  |
| Semantic x Ambiguity | -0.02 | 0.12 | -0.19 | 116 | 0.850 |  |
| Semantic x Unambiguous | 0.24 | 0.14 | 1.66 | 116 | 0.099 |  |
| AUT x Ambiguity | 0.89 | 2.60 | 0.34 | 116 | 0.732 |  |
| AUT x Unambiguous | 2.23 | 3.00 | 0.74 | 116 | 0.458 |  |
| RAT x Ambiguity | 0.05 | 0.11 | 0.45 | 116 | 0.652 |  |
| RAT x Unambiguous | -0.09 | 0.13 | -0.72 | 116 | 0.475 |  |
| Flanker RT x Ambiguity | -0.77 | 0.66 | -1.18 | 116 | 0.241 |  |
| Flanker RT x Unambiguous | -0.14 | 0.76 | -0.18 | 116 | 0.857 |  |
| Flanker Error Rate x Ambiguity | -0.56 | 0.28 | -2.03 | 116 | 0.044 | * |
| Flanker Error Rate x Unambiguous | -0.13 | 0.32 | -0.42 | 116 | 0.676 |  |

*Note.* Results of multiple imputation model of individual differences predicting ease of conceptual combination in younger adults. Model: with (imp, lmer(ease ~ 1 + vviq_z + semantic_z + aut_z + rat_z + flanker_rt_z + flanker_err_z + comboType + vviq_z:comboType + semantic_z:comboType + aut_z:comboType + rat_z:comboType + flanker_rt_z:comboType + flanker_err_z:comboType + (1|id), data = indivDiff_data))

**Table S5.**

*Correlations Among Individual Difference Measures.*

| Measure | | 1 | 2 | 3 | 4 | 5 | 6 | |
| --- | --- | --- | --- | --- | --- | --- | --- | --- |
| Younger Adults | |  |  |  |  |  |  | |
|  | | 1. VVIQ | - |  |  |  |  |  |
|  | | 2. Semantic | 0.073 | - |  |  |  |  |
|  | | 3. AUT | 0.051 | 0.073 | - |  |  |  |
|  | | 4. RAT | –0.176* | 0.182* | –0.101 | - |  |  |
|  | | 5. Flanker RT | 0.236** | –0.029 | 0.026 | –0.344*** | - |  |
|  | | 6. Flanker Error | –0.191* | 0.035 | –0.005 | –0.107 | –0.457*** | - |
| Older Adults | |  |  |  |  |  |  | |
|  | 1. VVIQ | - |  |  |  |  |  | |
|  | 2. Semantic | –0.065 | - |  |  |  |  | |
|  | 3. AUT | 0.156* | –0.028 | - |  |  |  | |
|  | 4. RAT | –0.014 | 0.400*** | –0.145* | - |  |  | |
|  | 5. Flanker RT | 0.008 | 0.166* | 0.038 | 0.047 | - |  | |
|  | 6. Flanker Error | 0.111 | 0.031 | –0.037 | 0.080 | 0.315*** | - | |

*Note.* All variables were z-scored. *p < .05. **p < .01. ***p < .001.

**Table S6. Full statistical model in all age groups.**

|  | **Estimate** | ***SE*** | ***statistic*** | ***df*** | ***p*** |  |
| --- | --- | --- | --- | --- | --- | --- |
| (Intercept) | -39.03 | 43.34 | -0.90 | 329 | 0.368 |  |
| Age Group (Younger) | -29.32 | 43.34 | -0.68 | 329 | 0.499 |  |
| Ambiguity (All vs. Ambiguous) | 35.66 | 34.94 | 1.02 | 329 | 0.308 |  |
| Unambiguous (Attributive vs. Relational) | 39.83 | 40.35 | 0.99 | 329 | 0.324 |  |
| AUT | -1.82 | 1.81 | -1.01 | 329 | 0.315 |  |
| Flanker RT | -0.91 | 0.64 | -1.41 | 329 | 0.160 |  |
| VVIQ | 0.11 | 0.07 | 1.58 | 329 | 0.116 |  |
| Semantic | 0.15 | 0.09 | 1.76 | 329 | 0.079 |  |
| RAT | 0.002 | 0.08 | 0.02 | 329 | 0.983 |  |
| Age Group x Ambiguity | -7.72 | 34.94 | -0.22 | 329 | 0.825 |  |
| Age Group x Unambiguous | 17.34 | 40.35 | 0.43 | 329 | 0.668 |  |
| Age Group x AUT | -1.03 | 1.81 | -0.57 | 329 | 0.568 |  |
| Age Group x Flanker RT | -0.51 | 0.64 | -0.79 | 329 | 0.428 |  |
| Age Group x VVIQ | -0.17 | 0.07 | -2.41 | 329 | 0.016 | * |
| Age Group x Semantic | -0.09 | 0.09 | -1.06 | 329 | 0.290 |  |
| Age Group x RAT | 0.09 | 0.08 | 1.21 | 329 | 0.226 |  |
| Ambiguity x AUT | 1.59 | 1.46 | 1.09 | 329 | 0.275 |  |
| Unambiguous x AUT | 1.71 | 1.68 | 1.02 | 329 | 0.310 |  |
| Ambiguity x Flanker RT | 0.76 | 0.52 | 1.46 | 329 | 0.146 |  |
| Unambiguous x Flanker RT | 0.54 | 0.60 | 0.90 | 329 | 0.371 |  |
| Ambiguity x VVIQ | -0.03 | 0.06 | -0.59 | 329 | 0.559 |  |
| Unambiguous x VVIQ | 0.003 | 0.06 | 0.05 | 329 | 0.962 |  |
| Ambiguity x Semantic | -0.03 | 0.07 | -0.46 | 329 | 0.646 |  |
| Unambiguous x Semantic | 0.11 | 0.08 | 1.30 | 329 | 0.194 |  |
| Ambiguity x RAT | 0.17 | 0.06 | 2.70 | 329 | 0.007 | ** |
| Unambiguous x RAT | -0.03 | 0.07 | -0.39 | 329 | 0.694 |  |
| Age Group x Ambiguity x AUT | -0.55 | 1.46 | -0.38 | 329 | 0.708 |  |
| Age Group x Unambiguous x AUT | 0.56 | 1.68 | 0.33 | 329 | 0.741 |  |
| Age Group x Ambiguity x Flanker RT | -0.85 | 0.52 | -1.63 | 329 | 0.104 |  |
| Age Group x Unambiguous x Flanker RT | -0.51 | 0.60 | -0.85 | 329 | 0.394 |  |
| Age Group x Ambiguity x VVIQ | -0.008 | 0.06 | -0.14 | 329 | 0.889 |  |
| Age Group x Unambiguous x VVIQ | -0.16 | 0.06 | -2.51 | 329 | 0.012 | * |
| Age Group x Ambiguity x Semantic | -0.02 | 0.07 | -0.25 | 329 | 0.801 |  |
| Age Group x Unambiguous x Semantic | 0.12 | 0.08 | 1.54 | 329 | 0.125 |  |
| Age Group x Ambiguity x RAT | -0.04 | 0.06 | -0.68 | 329 | 0.498 |  |
| Age Group x Unambiguous x RAT | -0.04 | 0.07 | -0.62 | 329 | 0.539 |  |

Results of regression model of individual differences predicting ease of conceptual combination across age groups. Age Group is contrast-coded (Younger = 1). Model: lmer(ease ~ 1 + age_group + comboType + aut_z + flanker_rt_z + vviq_z + semantic_z + rat_z + age_group:comboType + age_group:aut_z + age_group:flanker_rt_z + age_group:vviq_z + age_group:semantic_z + age_group:rat_z + comboType:aut_z + comboType:flanker_rt_z + comboType:vviq_z + comboType:semantic_z + comboType:rat_z + age_group:comboType:aut_z + age_group:comboType:flanker_rt_z + age_group:comboType:vviq_z + age_group:comboType:semantic_z + age_group:comboType:rat_z + (1|id), data = indivDiff_data)
